# Transmissible Endoplasmic Reticulum Stress Mediated by Extracellular Vesicles from Adipocyte Promoting the Senescence of Adipose-Derived Mesenchymal Stem Cells in Hypertrophic Obesity

**DOI:** 10.1155/2022/7175027

**Published:** 2022-08-05

**Authors:** Jia Fang, Li Li, Xingguo Cao, Han Yue, Wanying Fu, Yi Chen, Zhiwei Xu, Qiongrui Zhao, Jingge Zhao, Yuebo Wang, Wulong Liang

**Affiliations:** ^1^Clinical Research Service Center, Henan Provincial People's Hospital, Zhengzhou University People's Hospital, Henan University People's Hospital, Zhengzhou, Henan 450000, China; ^2^Stem Cell Research Center, Henan Key Laboratory of Stem Cell Differentiation and Modification, Henan Provincial People's Hospital; Zhengzhou University People's Hospital; Henan University People's Hospital, Zhengzhou, Henan 450000, China; ^3^School of Basic Medicine, Xinxiang Medical University, Xinxiang, Henan 453003, China; ^4^Department of Neurosurgery, The Fifth Affiliated Hospital of Zhengzhou University, Zhengzhou, Henan 450000, China

## Abstract

Hypertrophic obesity, characterized by an excessive expansion of subcutaneous adipocytes, causes chronic inflammation and insulin resistance. It is the primary feature of obesity in middle-aged and elderly individuals. In the adipose microenvironment, a high level of endoplasmic reticulum (ER) stress and changes in the extracellular vesicle (EV) composition of adipocytes may cause the senescence and restrained differentiation of progenitor cells of adipose, including adipose-derived mesenchymal stem cells (ASCs). In this study, a hypertrophic obesity mouse model was established, and the effects of adipocytes on the ER stress and senescence of ASCs were observed in a coculture of control ASCs and hypertrophic obesity mouse adipocytes or their derived EVs. The adipocytes of hypertrophic obesity mice were treated with GW4869 or an iron chelation agent to observe the effects of EVs secreted by adipocytes and their iron contents on the ER stress and senescence of ASCs. Results showed higher ER stress level and senescence phenotypes in the ASCs from the hypertrophic obesity mice than in those from the control mice. The ER stress, senescence phenotypes, and ferritin level of ASCs can be aggravated by the coculture of ASCs with adipocytes or EVs released by them from the hypertrophic obesity mice. GW4869 or iron chelator treatment improved the ER stress and senescence of the ASCs cocultured with EVs released by the adipocytes of the hypertrophic obesity mice. Our findings suggest that EV-mediated transmissible ER stress is responsible for the senescence of ASCs in hypertrophic obesity mice.

## 1. Background

Metabolic diseases caused by obesity are important public health problems in modern society. Subcutaneous adipose tissue (SAT) is the largest lipid depot of the body [1]. Adipose tissues expand through the recruitment of adipogenic progenitors and hypertrophic expansion of differentiated adipocytes that contribute to hypertrophic obesity [2]. However, the ability of adipocytes to expand and store excess calories is limited, causing obesity-associated metabolic complications [1]. Hypertrophic adipocytes result in an altered secretion profile characterized by increased levels of leptin and inflammatory cytokines and reduced level of adiponectin, which lead to chronic inflammation, increased oxidative stress, and endoplasmic reticulum (ER) stress [3]. Adipose tissues regulate metabolism, insulin resistance (IR), and the development of type 2 diabetes (T2D) through the secretion of extracellular vesicles (EVs) [4]. EVs are spherical vesicles with outer lipid bilayers and internal contents including RNA, proteins, and lipids [5]. In T2Ds, EVs released from adipocytes play an important role in intercellular communications between adipocytes and macrophages [6] and may affect the function of mesenchymal stem cells in adipose tissues.

Adipose-derived mesenchymal stem cells (ASCs) are adult stem cells, with self-renewal and multipotential differentiation capacities [7]. ASCs can be differentiated into adipocytes and are components of the lineage hierarchy of adipocyte progenitors [8, 9]. The senescence and differentiation inhibition of ASCs are more serious in individuals with obesity or metabolic diseases than in normal individuals [9], but the underlying mechanisms are unclear.

Growing evidence has shown that ER stress activation is a central feature involved in the pathogenesis of different diseases, including obesity-associated IR and diabetes [10]. ER stress is the consequence of a lack of control in the amount of poorly folded proteins inside the ER, resulting in a reduction in the protein synthesis and expression of various transcription factors. Cells upon ER stress can induce ER stress to other cells. This concept is defined as transmissible ER stress (TERS) [11]. EVs can be a contributory factor in transmitting ER stress in the tissue microenvironment [12].

The present study is aimed at elucidating the mechanisms by which ER stress transmitted from expanded adipocytes promotes senescence and inhibits the differentiation of ASCs in a mouse model with hypertrophic obesity. Results show that EVs accomplish these changes in part by trafficking iron, a regulator that causes senescence through its accumulation.

## 2. Results

### 2.1. High ER Stress Levels in Adipocytes and ASCs of Hypertrophic Obesity Mice

**Figure 1:**
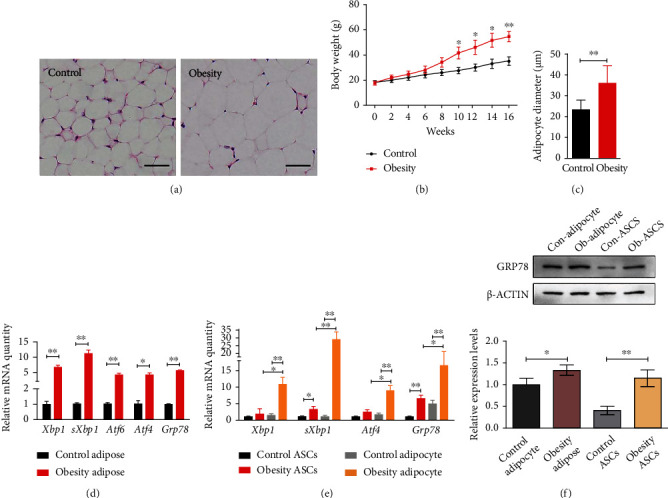
Higher ER stress levels in the adipocytes and ASCs of hypertrophic obesity mice. (a) HE staining of subcutaneous adipose tissue section of control and hypertrophic obesity mice. Bar = 50 *μ*m. (b) Mean subcutaneous adipocyte diameter of control and hypertrophic obesity mice. (c) Body weight of mice after control and hypertrophic obesity diet. (d) QRT-PCR analysis of the relative mRNA expression of ER stress-related genes in adipose of control and hypertrophic obesity mice. (e) QRT-PCR analysis of the relative mRNA expression of ER stress-related genes in adipocytes and ASCs. (f) Western blot and multiple quantifications of ER stress-related protein in adipocytes and ASCs of control and hypertrophic obesity mice.

**Figure 2:**
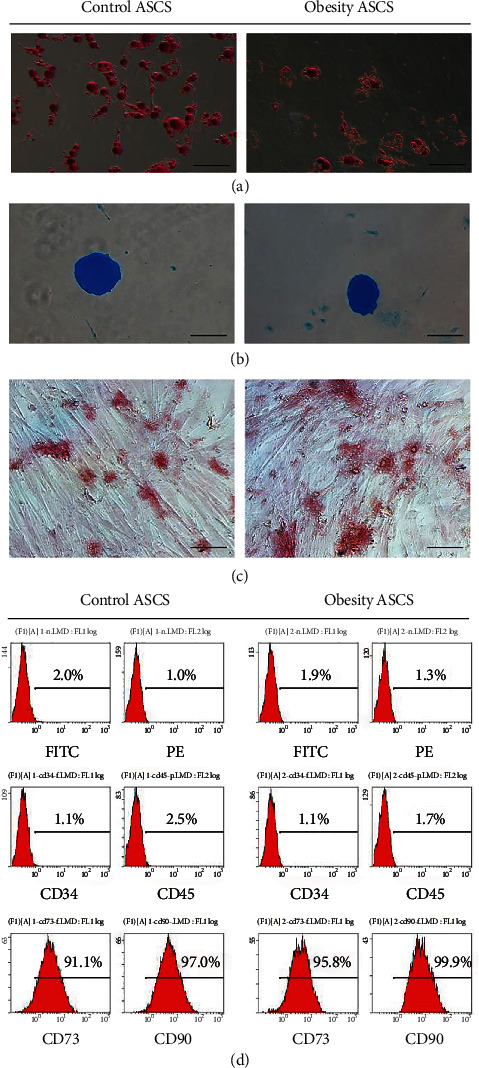
Identification of ASCs. (a) Oil red O staining for adipocyte differentiation of ASCs. Bar = 100 *μ*m. (b) Alcian blue staining for chondrocyte differentiation of ASCs. Bar = 200 *μ*m. (c) Alizarin red staining for osteoblast differentiation of ASCs. Bar = 50 *μ*m. (d) Characterization of ASC surface markers by flow cytometry.

The hypertrophic obesity murine model was fed a high-fat diet for 16 weeks to induce obesity. The mean subcutaneous adipocyte diameter was significantly larger in the hypertrophic obesity mice than in the control mice (Figures 1(a) and 1(c)). The body weights of the hypertrophic obesity mice were significantly higher than those of the control mice (Figure 1(b)). Analysis of subcutaneous adipose tissue samples revealed that the mRNA expression levels of ER stress markers (*Xbp1*, *sXbp1*, *Atf6*, *Atf4*, and *Grp78*) were significantly higher in the hypertrophic obesity mice than in the control mice (Figure 1(d)). To further investigate the contribution of each cell type to ER stress, we examined the mRNA expression and protein levels of ER stress markers of adipocytes and ASCs. Before this procedure, we isolated the ASCs and identified their differentiation ability. Isolated ASCs can differentiate into adipocyte (Figure 2(a)), chondrocyte (Figure 2(b)), and osteoblast (Figure 2(c)) in corresponding differentiation induction culture medium. The surface markers of ASCs were positive for CD73, CD90, and negative for CD34 and CD45 (Figure 2(d)). The expression levels of ER stress-related genes and GRP78 protein were significantly higher in the adipocytes than in the ASCs derived from the subcutaneous adipocytes of the hypertrophic obesity mice (Figures 1(e) and 1(f)).

### 2.2. Increased Senescent Phenotype in ASCs in Hypertrophic Obesity Mice

**Figure 3:**
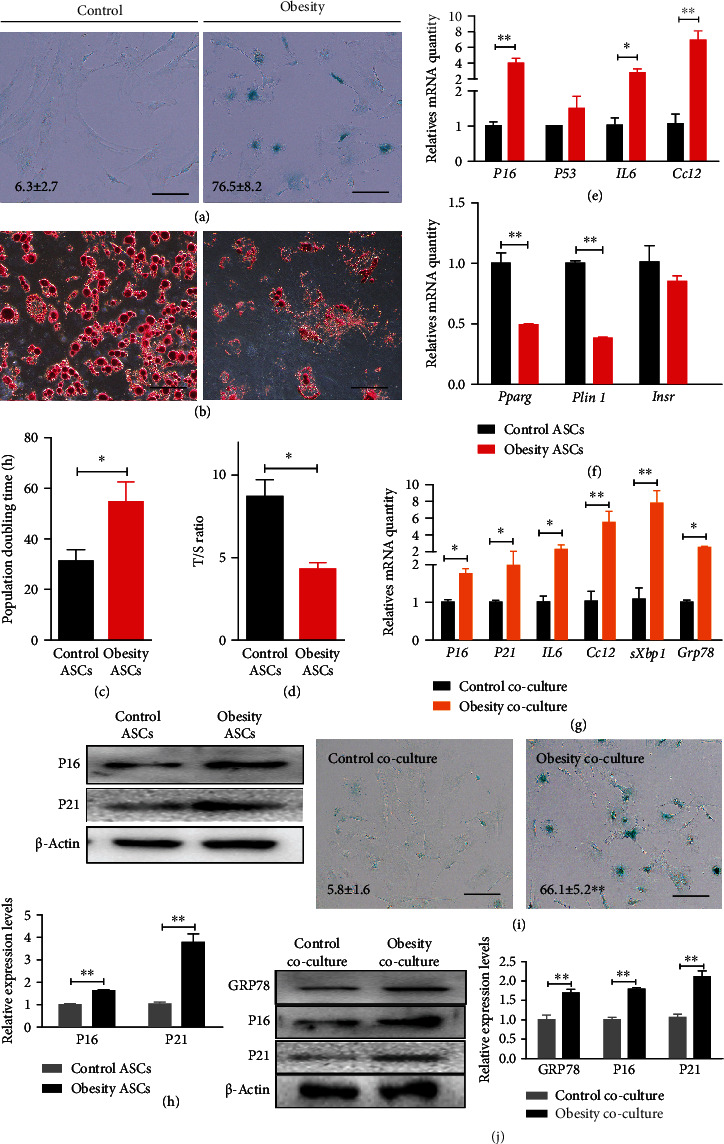
Increased senescent phenotype in ASCs in hypertrophic obesity mice. (a) SA-*β*-gal staining of control and hypertrophic obesity ASCs. Bar = 100 *μ*m. (b) Oil red O staining of adipogenic-induced control and hypertrophic obesity ASCs. Bar = 100 *μ*m. (c) Population doubling time of control and hypertrophic obesity ASCs. (d) Relative telomere length of control and hypertrophic obesity ASCs. (e) QRT-PCR analysis of the relative mRNA expression of senescent markers (*P16* and *P53*) and SASP-related markers (*IL6* and *Ccl2*) in control and hypertrophic obesity ASCs. (f) QRT-PCR analysis of the relative mRNA expression of adipogenic differentiation-related genes in adipogenic induced of control and hypertrophic obesity ASCs. (g) QRT-PCR analysis of the relative mRNA expression of senescence and ER stress-related genes in control and hypertrophic obesity adipocytes cocultured ASCs. (h) Western blot and multiple quantifications of senescent markers (P16 and P21) of control and hypertrophic obesity ASCs. (i) SA-*β*-gal staining of control and hypertrophic obesity adipocytes cocultured ASCs. Bar = 100 *μ*m. (j) Western blot and multiple quantifications of senescence and ER stress-related proteins in control and hypertrophic obesity adipocytes cocultured ASCs.

We previously reported that chronic ER stress increases with aging [13]. The aging phenotype of adipogenic precursor cells was detected in the hypertrophic obesity individual at high risk of T2D [9]. Therefore, we measured the senescence phenotype of the ASCs in the hypertrophic obesity mice. The positive rate of SA-*β*-gal staining was significantly higher in the ASCs from the hypertrophic obesity mice than in those from the control mice (Figure 3(a)). The results of Oil red O staining (Figure 3(b)) and adipocyte-specific genes (*Pparg*, *Plin1*, and *Insr*) expression detection (Figure 3(f)) indicated that the adipogenic differentiation potential of ASCs decreased in the hypertrophic obesity mice. The population doubling time (Figure 3(c)) increased, and the relative telomerase length T/S ratio (Figure 3(d)) decreased in the ASCs from the hypertrophic obesity mice. Senescent markers (*P16* and *P53*) and senescence-associated secretory phenotype (SASP) markers (*IL6* and *Ccl2*) also increased in the ASCs from the hypertrophic obesity mice (Figures 3(e) and 3(h)).

### 2.3. Adipocyte Induces ER Stress and Senescence in ASCs *In Vitro*

Considering that the ER stress levels in the adipocytes are higher than those in the ASCs from the hypertrophic obesity mice, we suspected that adipocytes transmit ER stress to ASCs. We cocultured normal ASCs with subcutaneous adipocytes from the hypertrophic obesity and normal control mice, respectively. ASCs were collected 48 h after coculture, and results showed that the mRNA expression and protein levels of senescent (*P16* and *P21*) and SASP (*IL6* and *Ccl2*) markers and ER stress-related genes (*sXbp1* and *Grp78*) (Figures 3(g) and 3(j)) were significantly higher in the ASCs cocultured with adipocytes from the hypertrophic obesity mice than in the ASCs cocultured with adipocytes from the control mice. The positive rate of SA-*β*-gal staining was significantly higher in the ASCs cocultured with adipocytes from the hypertrophic obesity mice than in the ASCs cocultured with adipocytes from the control mice (Figure 3(i)).

### 2.4. EVs from Obesity Adipocytes Promote TERS and Senescence in ASCs

**Figure 4:**
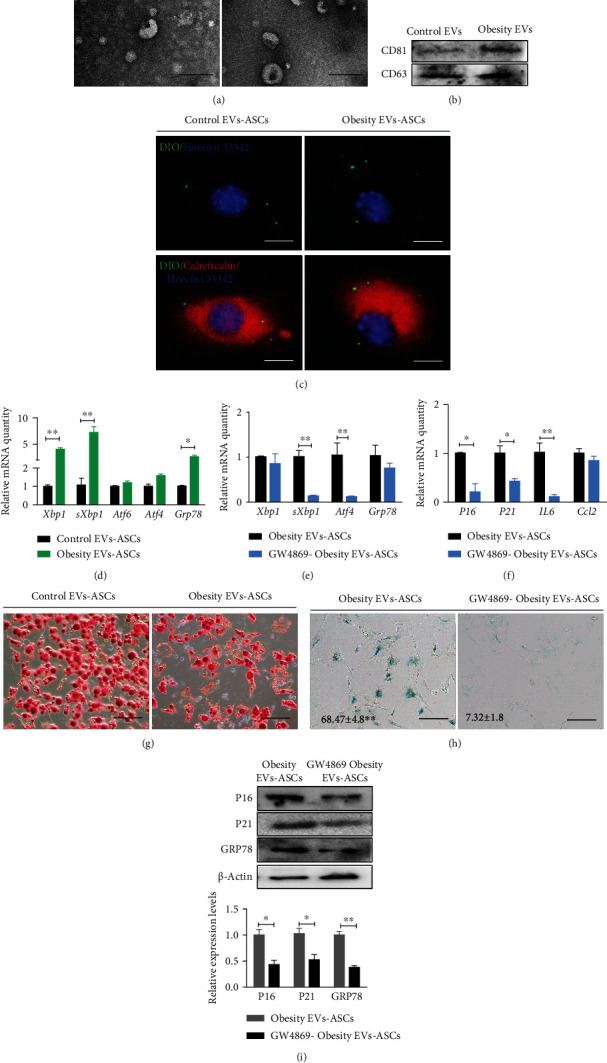
Effects of adipocyte-secreted EVs on ASCs senescence and ER stress. (a) Transmission electron microscopy showed that the EVs of control and hypertrophic obesity adipocytes presented with the typical morphology. Bar = 200 nm. (b) Western blot on EV markers for control and hypertrophic obesity adipocyte-secreted EVs. (c) Immunofluorescence staining of ER marker Calreticulin in ASCs pretreated with DiO-labeled EVs from control and hypertrophic obesity adipocytes. Bar = 50 *μ*m. (d) QRT-PCR analysis of the relative mRNA expression of ER stress-related genes in ASCs pretreated with EVs secreted by control and hypertrophic obesity adipocytes. (e, f) QRT-PCR analysis of the relative mRNA expression of ER stress and senescence markers in hypertrophic obesity adipocyte-derived EVs cocultured ASCs and GW4869 pretreated hypertrophic obesity adipocyte-derived EVs cocultured ASCs. (g) Oil red O staining of adipogenic-induced ASCs pretreated with EVs secreted by control and hypertrophic obesity adipocytes. Bar = 100 *μ*m. (h) SA-*β*-gal staining of hypertrophic obesity adipocyte-derived EVs cocultured ASCs and GW4869 pretreated hypertrophic obesity adipocyte-derived EVs cocultured ASCs. Bar = 100 *μ*m. (i) Western blot and multiple quantifications of P16, P21, and GRP78 in hypertrophic obesity adipocyte-derived EVs cocultured ASCs and GW4869 pretreated hypertrophic obesity adipocyte-derived EVs cocultured ASCs.

Cells can transmit ER stress to other cells in the microenvironment through EVs [12]. EVs in the supernatant culture of adipocytes from hypertrophic obesity and control mice were isolated and cocultured with control ASCs to explore the effects of EVs on the ER stress and senescence of ASCs. The morphology of the EVs was detected using transmission electron microscopy (Figure 4(a)). The protein markers for EVs (CD63 and CD81) were identified through western blot (Figure 4(b)). The EVs isolated from the adipocytes in the obese group had a higher protein content than the EVs from the adipocytes in the control group. EVs were stained with DIO before coculture with ASCs. By fluorescence microscope observation, EVs secreted by adipocytes could enter ASCs and locate in the region near the ER (Figure 4(c)). The mRNA expression levels of ER stress markers (*Xbp1*, *sXbp1*, *Atf4*, *Atf6*, and *Grp78*) were significantly higher in the ASCs treated with hypertrophic obesity adipocyte-derived EVs than in the ASCs treated with control adipocyte-derived EVs (Figure 4(d)). After 2 days of EV treatment, ASCs were placed in an adipocyte induction solution for 14 days. Oil red O staining showed that the adipogenic potential of the ASCs pretreated with EVs from the hypertrophic obesity adipocytes was lower than that of the ASCs pretreated with EVs from the control obesity adipocytes (Figure 4(g)).

To investigate the effect of adipocyte EVs on ASCs, adipocytes from the obesity mice were treated with 20 *μ*M GW4869, which can inhibit EV secretion, or DMSO for 12 h. Then, adipocytes were switched to normal serum-free medium for 12 h. EVs from the GW4869-pretreated adipocytes or control adipocytes were added to ASCs culture medium for 12 h. The mRNA and protein levels of ER stress (*Xbp1*, *sXbp1*, *Atf4*, and *Grp78*) (Figures 4(e) and 4(i)), senescent (*P16* and *P21*), and SASP (*IL6* and *Ccl2*) markers (Figures 4(f) and 4(i)) and the positive rate of SA-*β*-gal staining (Figure 4(h)) were lower in the ASCs cocultured with EVs derived from the GW4869-pretreated obesity adipocytes than in the ASCs cocultured with EVs derived from obesity adipocytes.

### 2.5. EVs Carrying Iron Promotes Iron Storage and Senescence in ASCs

**Figure 5:**
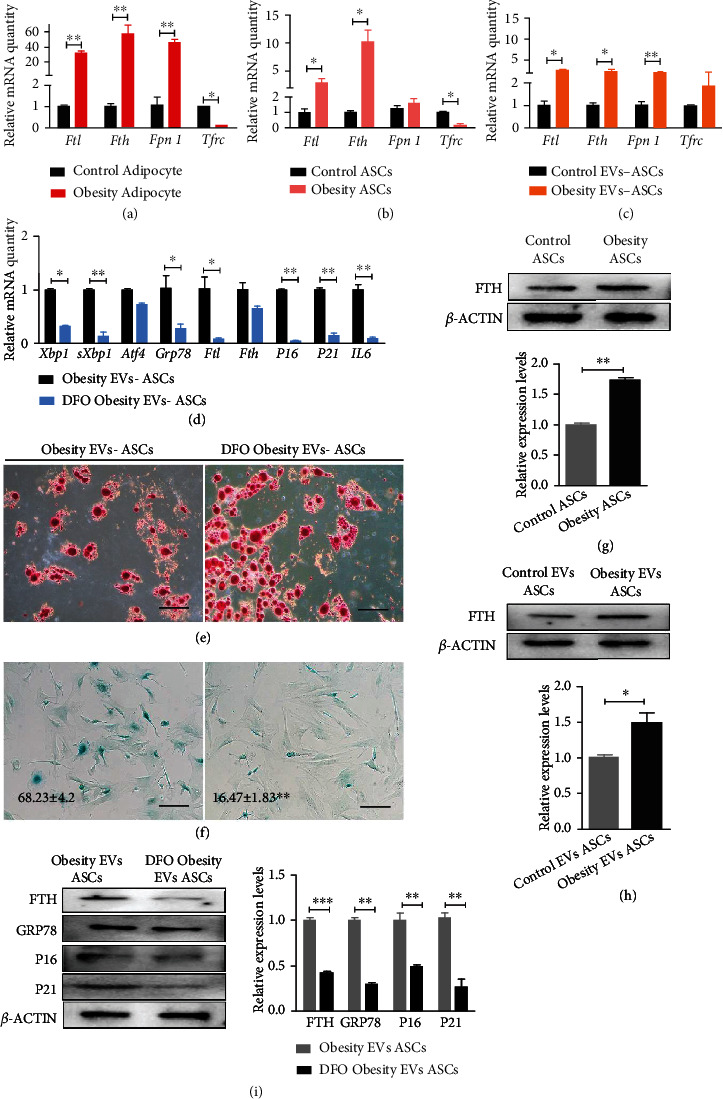
Effects of iron in adipocyte-derived EVs on ASC senescence and ER stress. (a) QRT-PCR analysis of the relative mRNA expression of iron-related genes in control and hypertrophic obesity adipocytes. (b) QRT-PCR analysis of the relative mRNA expression of iron-related genes in control and hypertrophic obesity ASCs. (c) QRT-PCR analysis of the relative mRNA expression of iron-related genes in control and hypertrophic obesity adipocyte-derived EVs cocultured ASCs. (d) QRT-PCR analysis of the relative mRNA expression of ER stress-related genes, senescence, and SASP-related genes in hypertrophic obesity adipocytes and DFO pretreated hypertrophic obesity adipocyte-derived EVs cocultured ASCs. (e) Oil red O staining of adipogenic-induced ASCs cocultured with EVs secreted by hypertrophic obesity adipocytes and DFO pretreated hypertrophic obesity adipocytes. Bar = 100 *μ*m. (f) SA-*β*-gal staining of ASCs cocultured with EVs secreted by hypertrophic obesity adipocytes and DFO pretreated hypertrophic obesity adipocytes. Bar = 100 *μ*m. (g) Western blot and multiple quantifications of iron-related protein in control and hypertrophic obesity ASCs. (h) Western blot and multiple quantifications of iron-related protein in control and hypertrophic obesity adipocyte-derived EVs cocultured ASCs. (i) Western blot and multiple quantifications of ER stress, senescence, and iron-related proteins in ASCs treated with EVs secreted by hypertrophic obesity adipocytes and DFO pretreated hypertrophic obesity adipocytes.

Iron accumulation is closely related to cell senescence [14]. The iron contents in the adipose tissues of obese individuals are significantly higher than those in the adipose tissues of individuals with normal weights [15]. Therefore, we examined the expression levels of iron metabolism-related genes in adipocytes and ASCs, respectively. The iron metabolism gene (*Ftl*, *Fth*, *Fpn1*, and *Tfrc*) and protein (FTH) levels were higher in the adipocytes and ASCs of the hypertrophic obesity mice than in those of the normal control mice (Figures 5(a), 5(b), and 5(g)).

Transport by EVs is an important route for iron metabolism. Coculture of adipocyte-derived EVs from the obese mice with EVs from the control mice could also improve the expression of iron metabolism-related genes and proteins in ASCs (Figures 5(c) and 5(h)). Adipocytes from the hypertrophic obesity mice were cultured in medium containing 50 *μ*M desferioxamine (DFO) or DMSO for 12 h to verify whether or not these cells can transmit ions through EVs and cause ASCs senescence. Then, the adipocytes were switched to the normal serum-free medium for 12 h. EVs from the DFO-pretreated adipocytes or control adipocytes were placed on ASCs culture medium for 12 h. ASCs were collected, and the expression levels of senescence and ER stress-related genes were measured. The expression levels of ER stress, iron metabolism, and senescence-related genes (Figure 5(d)) and proteins (Figure 5(i)) were significantly lower in the DFO-treatment group than in the control group. Oil red O staining and SA-*β*-gal staining showed that the adipogenic potential and senescence were lower in the ASCs cocultured with EVs derived from the obesity adipocytes pretreated with DFO than in the ASCs cocultured with EVs derived from the obesity adipocytes (Figures 5(e) and 5(f)).

## 3. Discussion

Adipose tissue expands its ability to store lipids through adipocyte proliferation and hypertrophy. Approximately 10% of adipocytes renew every year, and hypertrophic adipocytes renew more slowly. Furthermore, the number of subcutaneous adipocytes is unlikely to change after puberty [1]. Thus, hypertrophic obesity tends to occur in adults. Adipocyte hypertrophy leads to morbid obesity and increases the incidence of metabolic diseases, such as IR and T2Ds, which are prevalent age-related pathologies. Transplanting adipose precursors or ASCs improves metabolic status [16]. Thus, the proliferation or differentiation of adipose precursors or ASCs is important to maintain the metabolic function of adipose tissue. In hypertrophic obesity, SAT adipogenesis is impaired by the poor differentiation rather than the reduced number of mesenchymal progenitor/precursor cells [17]. The senescence of mesenchymal progenitor/precursor cells may be the cause of adipose differentiation failure and responsible for adipocyte hypertrophy in adult individuals [18]. To demonstrate the prevalence of this view on diet-induced adult obesity, we fed adult mice a high-fat diet to create hypertrophy obesity mouse model. Similar results were observed in the senescence and differentiation inhibition of ASCs in the obesity mouse model.

In line with the prolongevity effect of ER stress inhibitors, ER stress has been described as a typical feature of molecular aging and that it accelerates aging across species [19]. Evidence suggests that ER stress-induced unfolded protein response (UPR) is not only an outcome but also an inducer of cellular senescence [20]. The IRE1 and ATF6 branch of the UPR control SASP through NF-*κ*B signaling and regulate the flat morphology of replicative senescence cells [20, 21]. The uncontrollable aggregation of unfolded or misfolded proteins is induced by electron leakage and reactive oxygen species (ROS) accumulation increases with age and activates ER stress, which induces many age-related and metabolic diseases, such as T2D.

Given that ER is the main site for the assembly and secretion of adipokines, ER stress plays an important role in adipocytes [22, 23]. Metabolic stress caused by excessive fatty acids and toxic lipids, including ceramides and palmitates, exerts cytotoxic effects on the ER [24]. Free fatty acids directly affect the ordering of the ER membrane and blockage of the ER-specific Ca^2+^–ATPase pump and increase adipocyte ROS production, a pathogenic event that can inhibit ER–Ca^2+^ channels and induce ER stress [25]. ER stress is higher in the adipose tissues of mice and humans with obesity, and reducing ER stress improves IR in obesity models [26, 27]. The results of the present study confirm that ER stress increases not only in the adipocytes of obese mice but also in ASCs. We also observed that ER stress can be transferred from adipocytes to ASCs, similar to previously reported ER stress transfer in tumor microenvironment [11, 12, 28]. However, most published studies have suggested that cells acquire ER stress by soluble factors released from cancer cells, such as BMP2. We suggest that EVs from adipocytes are also mediators that cause ER stress transmission. Studies have reported that EVs can enter cells through the filopodia, and that endocytic vesicles with high efficiency are targeted for ER scanning and probably release cargoes [29]. EV-treated cells display a dilated ER and an increased ER stress level [12, 30], consistent with our observations.

In the present study, iron transfer through EVs from adipose cells triggered ER stress and senescence in ASCs. Iron has prooxidant properties owing to its redox activity. Therefore, iron plays vital roles in oxygen transport, electron transfer, and cell growth and differentiation. Iron accumulation can increase the aggregation of toxic proteins and induce oxidative stress and inflammation through the Fenton reaction, which lead to cellular damage and accelerate telomere shortening and senescence [31, 32]. The precise mechanisms by which ER stress is affected by changes in iron levels remains unclear. Previous studies have shown that iron chelators lower ER stress by blocking the phosphorylation of PERK and its downstream [33].

Iron is mainly found in the hemoglobin and myoglobin and present in trace amounts in enzymes, such as cytochrome, cytochrome oxidase, peroxidase, and catalase. Iron overload can activate an abnormal oxidative phosphorylation pathway in the mitochondria. Large amounts of ROS are produced during ATP production. ROS can oxidize unsaturated fatty acids on cell and organelle membranes and promote the formation of lipid peroxides, which directly or indirectly damage cell structure and function and ultimately lead to cell damage or ferroptosis [34]. Iron also regulates ferroptosis by forming enzymes involved in lipid peroxidation, such as lipoxygenase. Thus, iron input, storage, and circulation are closely related to ferroptosis. During ferroptosis, a large amount of accumulated lipid peroxides and lipid ROS are dispersed in the membrane and organelle membranes, such as mitochondrial, lysosome, and ER membranes, and destroy membrane structures [35]. The excessive accumulation of iron also occurs in senescent cells. The decrease in antioxidant capacity of senescent cells accelerates lipid peroxidation and aging of the body. This process is defined as ferrosenescence [36]. In the mitochondria, iron-mediated p53 inactivation can stimulate the iron–sulfur cluster assembly enzyme and promote iron accumulation. Mitochondrial p53 inactivation may lead to mitochondrial DNA damage. This vicious cycle destroys mitochondrial function and accelerates aging [37].

Iron overload is associated with high risk of neurodegenerative, metabolic, and other age-related diseases. Iron in excessive amounts leads to the impaired differentiation of adipose progenitor cells [38], weight gain, adipocyte hypertrophy, adipose tissue macrophage infiltration, and IR, all of which are phenotypes of diabetogenic adipocyte [38, 39]. However, iron reduction reverses these phenotypes [40, 41]. In the present study, senescence phenotypes, ER stress, and the adipogenic differentiation potential of ASCs can be alleviated by iron chelation agents in adipocytes. Thus, the iron content of adipocytes may affect the senescence and differentiation potential for ASCs in microenvironment.

The main transport mode of iron is dependent on transferrin and its receptor system. A recent study has reported that ferritin carrying iron can be transmitted via EVs. Exosome-mediated iron secretion is a protective mechanism against ferroptotic cell death [42]. In hypertrophic adipocytes with elevated stress levels, the amount of EVs released increases [43]. This iron output may be a self-protection mechanism. This study showed that increased iron content in the adipocytes indirectly increased stress in surrounding ASCs. Therefore, iron may also play a mediating role in individual energy metabolism through intercellular transmission. However, we only carried out in vitro experiments on this phenomenon. Thus, the transmission of EVs carrying iron in the microenvironment in vivo and the resulting ER stress transmission remain unknown. In addition, iron may not be the only factor that transfers ER stress. Other EV contents, such as miRNA, may be also transmit ER stress, which needs further experimental verification in the future.

## 4. Conclusion

**Figure 6:**
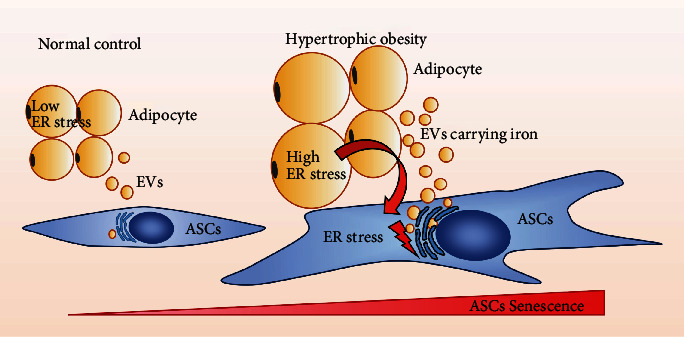
Mode of transmissible ER stress promoting ASC senescence. High level of ER stress in hypertrophic obesity adipocytes can be transferred to ASCs through iron carried by EVs. Senescence phenotype intensifies with increasing ER stress level in ASCs.

Hypertrophic adipocytes cause the aging and differentiation retardation of ASCs through transportable ER stress (Figure 6). The targeted reduction of EV secretion or iron output with hypertrophic adipocytes may contribute to the treatment of hypertrophic obesity and IR.

## 5. Methods and Materials

### 5.1. Cell Isolation and Culture

ASCs and adipocytes were isolated from the subcutaneous inguinal white adipose tissues of mice with collagenase type I solution (Roche Diagnostics, Switzerland). Adipocytes were obtained in the upper layer of the tissue digests. ASCs isolated from the stromal vascular fraction (SVF) in the bottom layer. ASCs and adipocytes were cultured in *α*-MEM or DM/F12 (Invitrogen, Carlsbad, CA) supplemented with 10% FBS (HyClone, USA), 2 mM L-glutamine, and 1% nonessential amino acids (Invitrogen) in a humid atmosphere with 5% CO_2_ at 37°C. Cells were dissociated every 2 days with trypsin- EDTA (Invitrogen, USA). ASCs were identified by flow cytometry for positive markers CD73, CD90, and negative markers CD34 and CD45.

### 5.2. Adipogenic Differentiation of ASCs In Vitro

For inducing adipogenic differentiation, 2 × 10^4^ cells were seeded into 12-well plates and induced by solution A and B alternate every three days for 14 days. Solution A was *α*-MEM consisting of 10% FBS, 1 *μ*M dexamethasone, 1 *μ*g/mL insulin, and 0.1 mM indomethacin. Solution B was *α*-MEM consisting of 10% FBS, 1 *μ*M dexamethasone, 1 *μ*g/mL insulin, 2 *μ*M rosiglitazone, and 0.1 mM indomethacin (Sigma-Aldrich, St. Louis, MO, USA). Adipogenic differentiation was identified by Oil red O staining.

### 5.3. Chondrogenic Differentiation of ASCs In Vitro

2 × 10^5^ ASCs were seeded into 35 mm suspension culture plates with 1 ml chondrogenesis differentiation medium. Chondrogenesis differentiation medium consists of *α*-MEM, 10% FBS, 40 ng/mL dexamethasone, 50 *μ*g/mL ascorbic acid, 50 *μ*g/mL L-proline, 1 mmol/L sodium pyruvate (all Sigma-Aldrich), insulin-transferrin-selenium X (Gibco, Carlsbad, California, USA), and 10 ng/mL transforming growth factor-*β*3 (PeproTech, Rocky Hill, NJ, USA) for 7 days. The chondrogenesis differentiation medium was changed every 2 days. Chondrogenic differentiation was identified by Alcian blue staining.

### 5.4. Osteogenic Differentiation of ASCs In Vitro

To induce osteogenic differentiation, 2 × 10^4^ cells were seeded into 12-well plates induced by *α*-MEM consisting of 10% FBS, 100 nmol/L dexamethasone, 30 *μ*g/mL ascorbic acid, and 10 mmol/L *β*-glycerophosphate (Sigma-Aldrich, St. Louis, MO, USA) for 7 days. Osteogenic differentiation was identified by Alizarin red staining.

### 5.5. Senescence-Associated *β*-Galactosidase Staining

ASCs were stained with a senescence-associated *β*-galactosidase (SA-*β*-gal) staining kit (Beyotime, China) according to the manufacturer's instructions. Fix ASCs for 15 min and wash with PBS for three times. Staining with the solution A, B, C, and X-gel mixed liquor for 10 h at 37°C. The positive rate of the SA-*β*-gal staining was the ratio of positive cells to the total number of cells in three different visual fields in each group.

### 5.6. Population Doubling Time (PDT) Determination

The population doubling time (PDT) of ASCs was estimated according to the formula PDT = [log 2/(log Nt − logN0)] × *t*. N0 indicates the number of seeded cells, Nt means the number of cells after *t* hours of culturing, and *t* stands for cell culturing time.

### 5.7. Telomere Length Assays

The genomic DNA of ASCs was extracted by using DNA Isolation Kit (Tiangen, China). The ratio of the telomere repeats copy number to single gene copy number (T/S) was determined using QRT-PCR in the CFX96 Real-Time PCR system. The QRT-PCR procedures were described as follows: predenaturation at 94°C for 10 min, followed by 39 cycles for 15 s at 94°C, and annealing for 1 min at 56°C. The telomere reaction mixture consisted of 2× Quantitect Sybr Green Master Mix, 100 nmol/L of Tel-F primer (CGGTTTGTTTGGGTTTGGGTTTGGGTTTGGGTTTGGGTT), and 900 nmol/L of Tel-R primer (GGCTTGCCTTACCCTTACCCTTACCCTTACCCTTACCCT). 36B4 was used as the loading control, with 36B4 primer (F: ACTGGTCTAGGACCCGAGAAG, R: TCAATGGTGCCTCTGGAGATT). Comparative CT values from QRT-PCR were used to draw the standard curve of control DNA samples of different concentrations. DNA quantitation of each gene can be calculated by using standard curve. The T/S ratio of each sample was calculated by dividing the average telomere ngDNA by the average 36B4 ngDNA value.

### 5.8. Quantitative Real-Time PCR Analysis

Trizol reagent (Takara, Japan) was used to extract the total RNA of ASCs according to the manufacturer's instructions. RNA was reverse transcripted into cDNA by using Reverse Transcriptase Reagent kit (Takara, Japan) according to the manufacturer's instructions. The quantitative real-time PCR (QRT-PCR) was carried on in the CFX96 Real-Time PCR system, and the QRT-PCR procedures were described as follows: predenaturation at 94°C for 5 min, followed by 39 cycles for 30 s at 94°C, and annealing for 30 s at 58°C and 30 s at 72°C for extending. 18S ribosomal RNA gene was used as the loading control. Comparative CT values from QRT-PCR were to measure relative gene expression. Primers are listed in Table 1.

### 5.9. EV Isolation and Uptake of DiO-Labeled EVs by ASCs

Conditioned medium from 2 × 10^6^ ASCs was collected and passed through a 0.8 *μ*m vacuum filter (Millipore, USA). According to the manufacturer's instruction, the EVs were collected with exoEasy Maxi Kit (Qiagen, Germany) to a final volume of 100 *μ*l and stored at -80°C for further analysis. EVs were identified by detecting tetraspanin protein CD81 and CD63 by western blots. The morphology of EVs was detected using transmission electron microscopy (Hitachi H7500 TEM, Japan).

EVs were incubated with DiO solution (Thermo, USA) for 20 min at 37°C. Excessive DiO was removed from Exosome Spin Columns (Thermo, USA). For immunocytochemistry, DiO-labeled EVs were added to the 1 × 10^5^ ASC culture media and incubated for 3 h at 37°C.

### 5.10. Immunofluorescence Staining

Cells were fixed in 4% paraformaldehyde at room temperature for 10 min. After washing with phosphate-buffered saline (PBS) for three times, cells were permeabilized for 15 min with 0.1% Triton-X 100 (Sigma, USA) at RT. Cells were blocked with 5% bovine serum albumin for 30 min and incubated with primary antibodies against Calreticulin (1 : 200; Abcam, UK), at 4°C for 16 h, washed three times with PBS. Cells were incubated with secondary antibodies for 1 h at 37°C in the dark. Nucleus counterstaining was performed with 1 *μ*g/mL Hoechst 33342 (Sigma-Aldrich), after three washing steps in PBS. Fluorescence images were obtained by IX73 fluorescence microscope (Olympus, Japan).

### 5.11. Western Blot Analysis

Total cell extracts were extracted with sodium dodecyl sulfate-polyacrylamide gel electrophoresis (SDS-PAGE) sample loading buffer. Cell proteins were resolved by SDS-PAGE, transferred to a polyvinylidene difluoride membrane, and probed for *β*-Actin (1 : 1000, Proteintech, USA), GRP78 CD63, CD81, and FTH (1 : 200; Bosterbio, USA), and P16 and P21 (1 : 200, Santa Cruz, USA). Horseradish peroxidase-conjugated anti-rabbit, anti-mouse (1 : 1000, Proteintech, USA) was used as a secondary antibody. Detection was performed using a Thermo Scientific Pierce enhanced chemiluminescence western blotting substrate (Thermo, USA). Results were analyzed by Bio-RAD ChemiDoc XRS gel imaging system (Bio-RAD, USA). The band density was determined by the Image J software (National Institutes of Health, Bethesda, USA).

### 5.12. Animals and Model

All procedures were performed in accordance with *Regulations for the Administration of Affairs Concerning Experimental Animals* in China and were approved by Life Science Ethics Review Committee of Zhengzhou University. Two-month-old male C57BL/6 mice were used according to Chinese Laboratory Animal Guidelines. There were 6 mice in each group. All mice were maintained under controlled light-dark cycle (12 h : 12 h lights cycle), temperature of 25 ± 1°C, and relative humidity 53 ± 2% and granted free access to standard dry chow and water. They were randomly assigned to two groups: control (low-fat diet contains 75.9% carbohydrate, 14.7% protein, and 9.4% fat) and hypertrophic obese (high-fat diet contains 20.8% carbohydrate, 18.3% protein, and 60.9% fat, in % kcal). All mice were fed in isolation cages for 16 weeks.

### 5.13. Statistical Analysis

One-way analysis of variance (one-way ANOVA) was used, and posttests were conducted using Newman–Keuls multiple range tests if *p* values were significant. Student's *t*-test was used in comparing only two pairs of data. All data were represented as mean ± SD, and statistical significance was expressed as follows: ^∗^*p* < 0.05; ^∗∗^*p* < 0.01; ^∗∗∗^*p* < 0.001. Each group had three independent samples. All data were analyzed using the GraphPad Prism software (CA, USA) and were representative of a minimum of three different experiments.

## Figures and Tables

**Table 1:** Primers used in quantitative real-time PCR analysis.

Gene	Forward primer (5′-3′)	Reverse primer (5′-3′)
*18S*	GGTCTGTGATGCCCTTAGATGTC	GAATGGGGTTCAACGGGTTAC
*Grp78*	TCATCGGACGCACTTGGAA	AACCACCTTGAATGGCAAGAA
*Xbp1*	AGCAGCAAGTGGTGGATTTG	GAGTTTTCTCCCGTAAAAGCTGA
*sXbp1*	GGTCTGCTGAGTCCGCAGCAGG	AGGCTTGGTGTATACATGG
*Atf4*	CTCAGACAGTGAACCCAATTGG	GGCAACCTGGTCGACTTTTATT
*Atf6*	CCCAAGCTCTCCGCATAGTC	TAAAATGCCCCATAACTGACCAA
*P16*	TTGCCCATCATCATCACCT	GGGTTTTCTTGGTGAAGTTCG
*P21*	CACAGCTCAGTGGACTGGAA	CCACCACCACACACCATAGA
*P53*	GGAAATTTGTATCCCGAGTATCTG	GTCTTCCACAGTGTGATGATGGTAA
*Il6*	AGTTGCCTTCTTGGGACTGATG	AACTCTTTTCTCATTTCCACGATTT
*Ccl2*	AGGTCCCTGTCATGCTTCTGG	AGGTGAGTGGGGCGTTAACTG
*Pparg*	GCCCTTTGGTGACTTTATGGAG	GCAGCAGGTTGTCTTGGATG
*Plin1*	GCAGAGGACCCAGAAGGCTC	GCCCATGCTGTGGTTTGC
*Insr*	CCACCAADAACTCGTGAAAGG	TGCACGCAGGAAAGAACCT
*Fth*	TGCCTCCTACGTCTATCTGTCTATG	CAGTCATCACGGTCTGGTTTCTT
*Ftl*	TCGTCAGAATTATTCCACCGAG	CCGATCAAAAAAGAAGCCCA
*Fpn1*	GCTGGTATTGATTTCAGTCTCCTT	GACCACAAACAAAAATCTGGTTG
*Trfc*	GAGCAGGGGAAATTACTTTTGC	CTGATGACTGAGATGGCGGA

## Data Availability

No data were used to support this study.

## Abbreviations

ER stress: Endoplasmic reticulum stress
EVs: Extracellular vesicles
ASCs: Adipose-derived mesenchymal stem cells
SAT: Subcutaneous adipose tissue
IR: Insulin resistance
T2D: Type 2 diabetes
TERS: Transmissible ER stress
SVF: Stromal vascular fraction
SA-*β*-gal: Senescence-associated *β*-galactosidase
PDT: Population doubling time
QRT-PCR: Quantitative real-time PCR
DFO: Desferioxamine
UPR: Unfolded protein response
ROS: Reactive oxygen species.
